# Salivary MicroRNAs: Diagnostic Markers of Mild Traumatic Brain Injury in Contact-Sport

**DOI:** 10.3389/fnmol.2018.00290

**Published:** 2018-08-20

**Authors:** Valentina Di Pietro, Edoardo Porto, Marco Ragusa, Cristina Barbagallo, David Davies, Mario Forcione, Ann Logan, Cinzia Di Pietro, Michele Purrello, Michael Grey, Douglas Hammond, Vijay Sawlani, Aron K. Barbey, Antonio Belli

**Affiliations:** ^1^Neurotrauma and Ophthalmology Research Group, Institute of Inflammation and Ageing, University of Birmingham, Birmingham, United Kingdom; ^2^National Institute for Health Research Surgical Reconstruction and Microbiology Research Centre, Queen Elizabeth Hospital, Birmingham, United Kingdom; ^3^Beckman Institute for Advanced Science and Technology, University of Illinois at Urbana–Champaign, Urbana, IL, United States; ^4^BioMolecular, Genome and Complex Systems BioMedicine Unit (BMGS), Section of Biology and Genetics G Sichel, Department of Biomedical Sciences and Biotechnology, University of Catania, Catania, Italy; ^5^IRCCS Associazione Oasi Maria S.S., Institute for Research on Mental Retardation and Brain Aging, Troina, Italy; ^6^School of Sport and Exercise, University of East Anglia, Norwich, United Kingdom

**Keywords:** concussion, mild traumatic brain injury, biomarkers, saliva, microRNA

## Abstract

Concussion is difficult to diagnose, particularly when symptoms are atypical or late in presenting. An accurate and timely initial assessment is crucial for clinical management. Cerebral spinal fluid (CSF) and blood markers of traumatic brain injury show promising results but their clinical applicability in concussion has significant limitations. In the study, we explored saliva as a new source of biomarkers of concussion. Saliva samples of concussed players were collected after 48–72 h from concussion and analyzed by high-throughput technologies. A discovery group of 10 concussed rugby professional and semiprofessional athletes and 10 non-concussed matched controls was used for the analysis of 92 inflammatory proteins by the Proseek-Multiplex-Inflammation technology. In addition, saliva samples of 6 concussed and 6 non-concussed athletes were used to screen 800 human microRNAs (miRNAs) by the Nanostring Technology. The results were then validated by RT-qPCR in an enlarged cohort (validation group) comprising 22 concussed athletes. Results showed, no significant variations of the 65 inflammatory proteins detected in saliva between groups but 5 microRNAs, miR-27b-3p (*p* = 0.016), let-7i-5p (*p* = 0.001), miR-142-3p (*p* = 0.008), miR-107 (*p* = 0.028), miR-135b-5p (*p* = 0.017) significantly upregulated in concussed athletes. Univariate ROC curve analysis showed that the differentially expressed miRNAs could be considered good classifiers of concussion. Further analyses showed significant correlation between these microRNAs and Reaction Time component of the ImPACT concussion assessment tool. In addition, biocomputation analysis predicted the involvement of these microRNAs in important biological processes that might be related to trauma, such as response to hypoxia, cell death, neurogenesis, axon repair and myelination. Ease of access and non-invasiveness of saliva samples make these biomarkers particularly suitable for concussion assessment.

## Introduction

There is growing concern that we may be facing a “concussion epidemic” in sport, military and recreational activities in general with significant associated unrecognized morbidity. Concussion, or mTBI (Harmon et al., 2013) can be defined as a transient impairment of neurological function after mechanical injury and affects up to 3.8 million participants in sports of all kind in the United States alone (Maroon et al., 2014). However, this injury is also common in the military (DeKosky et al., 2010) and has been described as the signature injury of the recent conflicts in Afghanistan and Iraq. The true incidence of mTBI is difficult to establish and is likely to be far greater than reported figures because type of injury is routinely under-recognized, trivialized and under-reported. This is partially due to the spontaneous resolution of symptoms in most patients even in the absence of medical care.

However, many studies have demonstrated that individuals who sustain one concussion are potentially more susceptible than others, especially if the new injury occurs before the symptoms from the previous concussion have completely resolved (Tavazzi et al., 2007; Vagnozzi et al., 2007, 2008). Importantly, repeated concussion is associated with depression (Vos et al., 2017) and chronic neurodegenerative conditions in later life, e.g., Parkinson’s disease, motor neuron disease and CTE (Omalu, 2014; Gaetz, 2017).

One of the main challenges faced by medical practitioners is the lack of objective parameters to support the diagnosis of concussion and guide return-to play-decisions following the injury (McCrory et al., 2017).

Currently, the vast majority of concussion assessment tools are indirect measures of the response to trauma. A concussion does not typically cause structural injury to the brain, thus neuroimaging tests such as MRI scan or CT scans are used primarily to rule out a more serious injury, but are not able to exclude the presence of mTBI currently.

Over the last few years, the measurement of biomarkers in biofluids has received growing attention. Serum/plasma and CSF are most studied biofluids, but it is possible that some central nervous system (CNS)-derived proteins are eventually excreted into body fluids other than CSF and blood. The presence of the axonal protein Tau in saliva, for example, was demonstrated using mass spectrometry (Shi et al., 2011). In addition, the same research group has also detected Parkinson-related α-synuclein and DJ-1 in this body fluid (Devic et al., 2011).

However, the relationship between salivary concentrations of these proteins and processes within the CNS is far from clear and no conclusive data on disease association have been reported so far.

Saliva is an important physiologic fluid, containing a highly complex mixture of substances, and is rapidly gaining interest for novel approaches to diagnosis, prognosis and management of patients with either oral or systemic diseases. In addition, it is easily collected and stored, and is ideal for POC devices.

In this study, we sought to identify saliva biomarkers from a well-characterized cohort of contact sport-professional and semiprofessional athletes with a view to developing a non-invasive, objective test to support clinical decision-making in sport and military medicine.

## Materials and Methods

### Study Approval

Study participants were recruited through the Surgical Reconstruction and Microbiology Research Centre (SRMRC), based at Queen Elizabeth Hospital of Birmingham (United Kingdom), as part of the ReCoS (The REpetitive COncussion in Sport). This study was carried out in accordance with the recommendations of the University of Birmingham Research Ethics Committee. The protocol was approved by that same ethical committee together with peer review by the National Institute of Health Research Centre for Surgical, Reconstruction and Microbiological Research Centre (NIHR SRMRC – Ethics Ref. 11-0429AP28). All subjects gave written informed consent in accordance with the Declaration of Helsinki.

Male athletes aged 16–65 years, participating in professional and semi-professional Rugby who have been positively diagnosed as having a concussion along with a normal neurological objective examination at assessment, were enrolled in this study. Individuals who require hospital admission after initial assessment for their TBI, presenting intracranial blood, brain tissue injury, or non-TBI related pathologies on initial CT/MR scan, any history of neurodegenerative pathology or history of chronic alcohol or drug abuse were excluded. In addition, age matched controls, who have not received any concussion in the previous 3 months, were recruited.

### Sample Collection

Saliva samples from a total of 20 subjects (discovery group) were analyzed. In particular, saliva samples of 10 concussed and 10 non-concussed athletes were used to screen proteins and 6 concussed and 6 non-concussed athletes for microRNA analysis. Saliva of concussed players was collected 48–72 h after a concussion, certified by the attending enhanced care team in accordance with the current protocol for the relevant sport, was confirmed. Samples from the non-concussed players were collected at rest before the training sessions. The athletes would not have done strenuous exercise since the day before at least.

From these data, we calculated the sample size needed for validation in a larger cohort of patients with alpha = 0.05 and power = 0.9. The sample size required was 29 subjects based on the most variant miRNA identified in the discovery group.

A second set of samples (validation group) was obtained from a total of 22 concussed and 10 non-concussed players. Samples were collected at the same time point.

Clinical, demographic (**Table 1**), neuropsychometric and imaging parameters were collected for each subject including, among others, parameters forming part of the SCAT3 (e.g., symptoms inventory, injury data, balance errors and immediate memory and recall tests), concussion history, the ImPACT (approved computerized neuropsychometric suite for sport concussion), and the WAIS-IV symbol search.

**Table 1:** Clinical and demographic characteristic of the study subjects included in the analysis of microRNA.

	Study group	Number of samples	Age			Gender	Days elapsed from concussion			Number of total professional career concussions		
			Mean	*SD*	Range	M/F	Mean	*SD*	Range	Mean	*SD*	Range
Discovery group	Controls	6	30	3.8	(22–33)	6/0				13.3	14.5	(0–5)
	Concussion	6	24.5	2.5	(21–28)	6/0	2.8	0.4	(2–3)	9.6	12.9	(0–4)
Validation Group	Controls	10	25.3	2.6	(20–28)	10/0				1.4	1.3	(0–4)
	Concussion	22	23.3	3.4	(17–31)	22/0	3.5	1.4	(2–5)	3.4	2	(0–8)

Saliva samples were also collected from an additional group consisting of 12 concussed athletes at later time points (>120 h).

### Sample Processing

Five ml of saliva were collected in a 50 ml sterile plastic universal container tube kept on ice for no more than 30′. Samples were then centrifuged at 2600 × *g* for 15′ at 4°C. Saliva was then divided into aliquots and to each ml of saliva, 1 ml aprotinin (stock 10 mg/ml), 3 ml Na3VO4 (stock 400 mM) and 10 ml PMSF (stock 10 mg/ml) was added for the analysis of 92 inflammatory proteins. RNase inhibitor (500 units/mL) was added for the analysis of miRNAs.

Samples were stored at -80°C until analysis.

### Proximity Extension Assay

The Proseek Multiplex Inflammation I (Olink Bioscience, Uppsala, Sweden) was used to perform the multiplex proximity assay according to the manufacturers protocol (Olink Bioscience, Uppsala, Sweden). Briefly, 1 μl of human saliva together with 3 μl of mix containing antibodies labeled with corresponding DNA oligonucleotides was incubated over night at 8°C. Following this, 96 μl of extension mix containing proximity extension assay enzymes and PCR reagents were added. Following a 5′ incubation plates were placed on the thermal cycler for 17 cycles of DNA amplification. The 96.96 Dynamic Array IFC (Fluidigm, CA, United States) was primed according to the manufacturer’s instructions. In a separate plate, 7.2 μl of detection mix and 2.8 μl of samples were mixed together and from this, 5 μl was loaded into the primed 96.96 Dynamic Array IFC. The specific primer pairs for the 92 inflammatory proteins (**Supplementary Table S1**) were loaded into the 96.96 dynamic array and the protein expression program activated in the Fluidigm Biomark reader as per Proseek instructions. Further details about detection limits, reproducibility and validations can be found at the Olink webpage^1^.

### Data Analysis

Data were transferred from the Biomark reader to Olink Wizard for GenEx software (Olink). From there, presented as NPX on a linear scale with high NPX corresponding to high protein concentration. Data were checked for normal distribution and *t*-test analysis was performed to compare the two groups (concussed vs. non-concussed athletes). Finally, *p*-values were adjusted for multiple testing through the False Discovery Rate method of correction (FDR).

### RNA Isolation

Total RNA was isolated from 400 μl of saliva by using Qiagen miRNeasy Mini Kit (Qiagen, GmbH, Hilden, Germany), according to Qiagen Supplementary Protocol for purification of RNA (including small RNAs) from serum or plasma (Ragusa et al., 2015). Finally, RNA was eluted in 200 μl RNAse-free water and subsequently precipitated by adding 20 μg glycogen, 0.1 volumes 3 M sodium acetate and 2.5 volumes ice cold 100% ethanol. After incubation at -80°C overnight, RNA was centrifuged and washed twice in ice cold 75% ethanol and resuspended in 7 μl RNAse-free water. RNA was quantified using a NanoDrop^®^ ND-1000 spectrophotometer (Thermo Scientific, Wilmington, DE, United States). An Agilent 2100 Bioanalyzer (Santa Clara, CA, United States) was used to detect the size distribution of total RNA, as well as determine the quality of the RNA. .

### Circulating miRNA Profiling Through NanoString Technology

Expression profile of miRNAs from saliva was performed through NanoString technology by using nCounter Human v3 miRNA Expression Assay Kits (NanoString Technologies) in an nCounter FLEX (Prep Station and Digital Analyzer) (NanoString Technologies), according to manufacturer instructions. Profiling was performed on 6 concussed and 6 non-concussed athletes. Three μl (approximately 150 ng) of total RNA were used for sample preparation. Data analysis was performed through nSolver 2.6 software. nCounter “normalized counts” were obtained from the raw counts from each hybridization normalized to the internal positive controls (to account for slight differences in assay efficiencies), and to three most stable reference miRNAs (to account for RNA amount differences), according to nSolver analysis software protocol. MiRNAs used as endogenous controls were selected through GMN method: we computed Pearson correlation between the count means for each lane and the counts of each miRNA, identifying those miRNAs whose expression was closer to the count mean of the cartridge (miR-23a-3p, miR-29b-3p, and miR-148b-3p) (Cirnigliaro et al., 2017). Statistical analysis was performed through SAM^2^, using a *p*-value based on 100 permutations; imputation engine: *K*-nearest neighbors (10 neighbors); FDR < 0.05.

Heat map of expression profiles was generated by plotting the fold changes calculated as the ratio between the normalized counts of each sample and the mean of normalized counts of all samples. By using Multi Experiment Viewer (MeV 4.9), we generated the sample clustering through hierarchical clustering approach by selecting Manhattan distance metric.

### Single TaqMan Assays

Twenty-one differentially expressed miRNAs were chosen from the arrays as potential candidate biomarkers of concussion. These candidates were used to validate the data in an enlarged cohort of 22 concussed athletes and 10 non-concussed athletes (validation group). Saliva was analyzed by single TaqMan assays (Applied Biosystems, Life Technologies^TM^). Samples were extracted as described above, retrotranscribed (Applied Biosystems, Life Technologies^TM^) and RT-qPCR analysis was performed in a Bio-Rad iQ5 Real-time PCR Detection System (Bio-Rad, CA, United States). Expression fold changes were calculated by the 2^-ΔΔCT^ method by using miR-23a-3p and miR-148b-3p as reference genes.

### miRNA Target Computational Analysis

We retrieved the experimentally validated targets of the Taqman confirmed DE-miRNAs (differentially expressed miRNA) from Tarbase^3^ and miRTarbase^4^ by selecting data just from highly confident experiments (e.g., luciferase or immunoprecipitation assays). We considered for downstream analysis all targets from both tools. Gene ontology enrichment of these targets was computationally analyzed by using FatiGo tool from Babelomics 4 suite ^5^ and DAVID Bioinformatics Resources 6.8^6^. Statistical over-representation was calculated by using Fisher’s exact test; Benjamini and Hochberg FDR Correction; *p* ≤ 0.05. The outputs of these analyses were compared and we reported just the matching gene ontologies. The tissue abundance of DE miRNAs was analyzed by screening the Human miRNA tissue atlas^7^.

### ImPACT and WAIS-IV Tests

The ImPACT test was designed in the early 1990s to assess concussed players of the National Football League (Maroon et al., 2014).

Nowadays, it is one of the most commonly used FDA (food and drug administration) approved neurocognitive tests in sport concussion (Covassin et al., 2009). The ImPACT includes a medical history and self-reported concussion symptoms questionnaire and neurocognitive tests. The neurocognitive tests are divided into six modules. The results of these modules score the following parameters: verbal memory, visual memory, visual-motor processing speed, reaction time and impulse control. The results are shown as row score and percentile. In the analysis of the data, age, gender, learning disability and level of education are taken into consideration. Another test to measure the cognitive abilities is the WAIS-IV. The WAIS-IV is compound of 10 tasks. Among these, the symbol search has demonstrated utility in the assessment of mTBI. Here, participants have to identify identical symbols in two rows within 2 min and errors of commission and omission are included to form a composite score. The NAB is a widely utilized clinically validated set of tests in TBI management. The digit span and reverse digit span numerical memory assessments are key components of this. These two tests give information regarding processing speed and working memory, respectively.

### Statistical Analysis

A non-parametric test (Mann–Whitney *U* test) was used to compare the level of miRNAs in the two independent groups. A *p*-value < 0.05 was accepted as significant.

In addition, a Receiver Operating Characteristic (ROC) analysis was utilized to calculate sensitivity and specificity of each biomarker in diagnosing concussion, expressed as AUC. Multivariate ROC curve was calculated by using MetaboAnalyst^8^.

Using a Spearman correlation analysis miRNA levels were also correlated with age, ImPACT and WAIS-IV.

An additional test was applied to compare miRNA expression at different time points, 48–72 h and >120 h, of concussed athletes with non-concussed athletes. The data were checked for normal distribution and transformed to perform parametric tests. Comparisons across groups at each time were performed by the one-way analysis of variance and Tukey’s *post hoc* test on transformed data.

All statistical analyses were carried on SPSS v.22 (IBM).

## Results

### Proseek Multiplex Analysis

**FIGURE 1:**
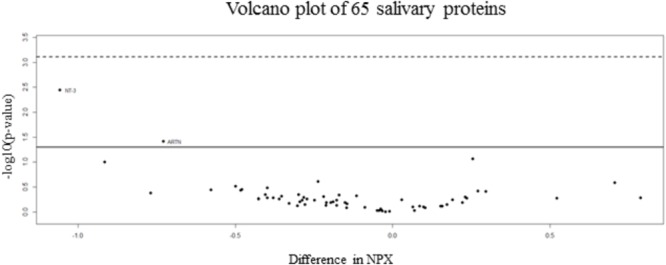
Volcano plot of salivary inflammatory proteins. The volcano plot visualizes the *p*-value (*y*-axis) and difference in NPX (*x*-axis) for all 65 analyzed proteins. Proteins on the positive *x*-axis have higher NPX-values in the non-concussed group and proteins on the negative *x*-axis have higher NPX-values in the concussed group. The solid line indicates an unadjusted *p*-value of 0.05 and the dashed line indicates approximated adjusted *p-value* (FDR) of 0.05.

92 human proteins were analyzed in saliva samples using the Proseek Multiplex Inflammation I panel. All samples met the quality control criteria and 65 of the 92 analyzed proteins were detected in all saliva samples. *t*-Test was applied for proteins detected and results showed no proteins significantly and differentially expressed between two groups. Volcano plot is represented in **Figure 1**. A full list of the 65 proteins detected in saliva together with fold changes and standard deviations among groups is also presented in **Supplementary Table S2**.

### Nanostring Profiling

Among the 800 microRNAs analyzed by nCounter NanoString in saliva of concussed and non-concussed athletes, 21 miRNAs were selected as differentially expressed across the two populations: hsa-let-7c-5p, hsa-let-7i-5p, hsa-miR-15b-5p, hsa-miR-16-5p, hsa-miR-20a-5p+hsa-miR-20b-5p, hsa-miR-24-3p, hsa-miR-27b-3p, hsa-miR-29a-3p, hsa-miR-29c-3p, hsa-miR-30a-5p, hsa-miR-107, hsa-miR-135b-5p, hsa-miR-142-3p, hsa-miR-148a-3p, hsa-miR-181a-5p, hsa-miR-199b-5p, hsa-miR-221-3p, hsa-miR-324-5p, hsa-miR-424-5p e hsa-miR-652-3p. Results showed a significant upregulation for all the miRNAs mentioned above.

**FIGURE 2:**
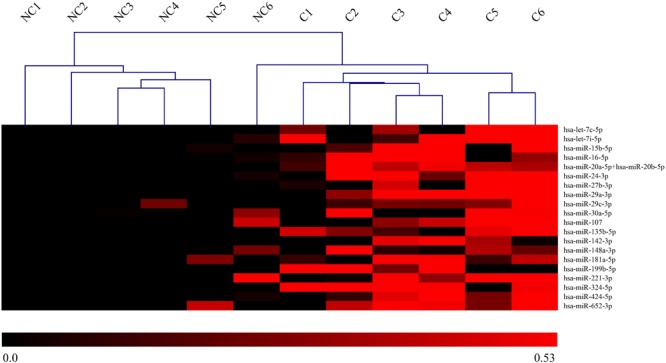
Heat-map of DE-miRNAs in saliva of concussed and non-concussed athletes. Heat-map of the miRNAs differentially expressed in saliva of concussed and non-concussed athletes. The values of log2 fold changes for each miRNA are color coded, as shown in the colored bar. Sample clustering obtained through hierarchical clustering approach is shown. NC, non-concussed athletes; C, concussed athletes.

A heat-map is represented in **Figure 2**.

### Single TaqMan Assay

**FIGURE 3:**
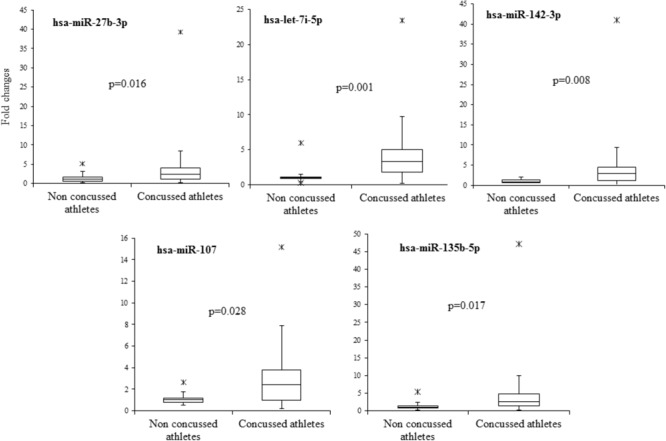
Boxplot of the 5 candidate miRNA biomarkers. Boxplot of relative expression of the five microRNAs which showed a significant upregulation (*p* < 0.05) in the validation group and assessed by RT-PCR. A non-parametric test (Mann–Whitney *U* test) was used to compare the level of microRNAs in the two independent groups.

**FIGURE 4:**
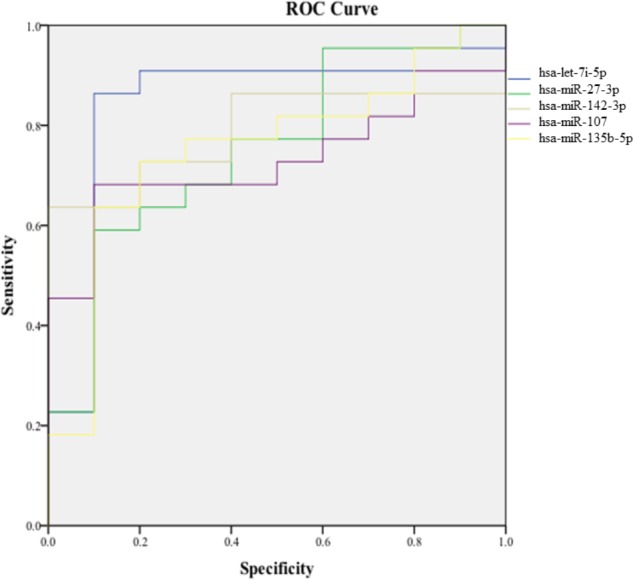
Area under the curve of the five candidate miRNA biomarkers. Receiver-operating characteristic (ROC) curve and corresponding area under the curve (AUC) for biomarkers identified in the validation cohort.

**FIGURE 5:**
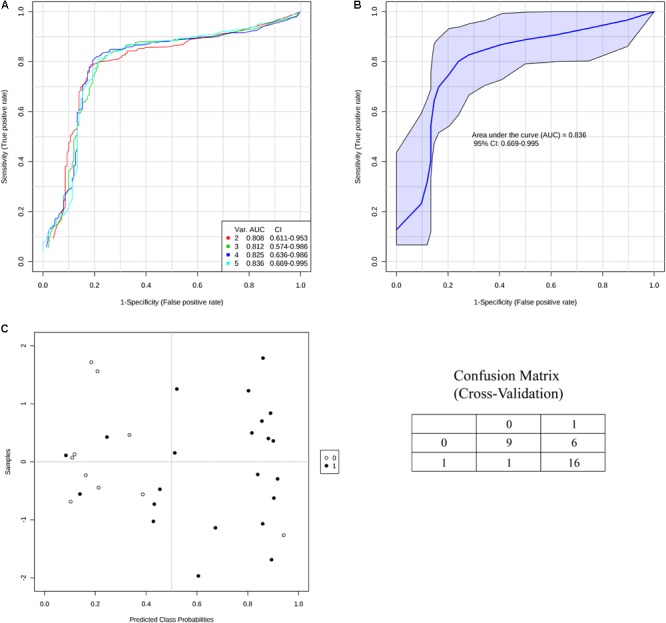
Multivariate ROC Curve analysis. **(A)** Four models of multivariate ROC curves, each one built on a combination of 2, 3, 4, or 5 DE miRNAs. **(B)** Five miRNA model ROC curve with confidence intervals highlighted in blue. **(C)** Sample classification plot and confusion matrix generated from the five miRNA model ROC curve.

In order to validate these findings, we subsequently tested the expression of the 21 selected miRNAs in a separate and independent group, composed of 22 concussed athletes and 10 matched non-concussed athletes. Among these candidate biomarkers for concussion, 5 were significantly upregulated in the validation group. Specifically, miR-27b-3p (*p* = 0.016), let-7i-5p (*p* = 0.001), miR-142-3p (*p* = 0.008), miR-107 (*p* = 0.028), miR-135b-5p (*p* = 0.017) confirmed the results obtained by Nanostring analysis (**Figure 3**). AUCs for miR-27b-3p (AUC, 0.755; 95% CI, 0.575–0.934), let-7i-5p (AUC, 0.845; 95% CI, 0.681–1), miR-142-3p (AUC, 0.791; 95% CI, 0.634–0.948), miR-107 (AUC, 0.732; 95% CI, 0.565–0.904), miR-135b-5p (AUC, 0.755; 95% CI, 0.573–0.936) are shown in **Figure 4**. We computed four models of multivariate ROC curves, each one built on a combination of 2, 3, 4, or 5 DE miRNAs, respectively, and compared their AUCs (**Figure 5**). The “5 miRNA” model showed the highest AUC: 0.836 (CI: 0.669–0.995). However, the comparison among confusion matrixes of univariate curves and “5 miRNA” model curve did not show an improvement of diagnostic performance for multivariate ROC curve, thus confirming let-7i-5p as the best performing biomarker of mTBI (**Table 2**).

**Table 2:** Comparison of performances of univariate and multivariate ROC curves.

	let-7i-5p	miR-142-3p	miR-107	miR-27b-3p	miR-135b-5p	Multivariate
Sensitivity	0.8636	0.7273	0.6818	0.6818	0.7273	0.7273
Specificity	0.1	0.2	0.1	0.3	0.2	0.1
Precision	0.6786	0.6667	0.625	0.6818	0.6667	0.64
Negative predictive value	0.25	0.25	0.125	0.3	0.25	0.1429
False positive rate	0.9	0.8	0.9	0.7	0.8	0.9
False discovery rate	0.3214	0.3333	0.375	0.3182	0.3333	0.36
False negative rate	0.1364	0.2727	0.3182	0.3182	0.2727	0.2727
Accuracy	0.625	0.5625	0.5	0.5625	0.5625	0.5313

### MiRNA Targets and Gene Ontology Analysis

**FIGURE 6:**
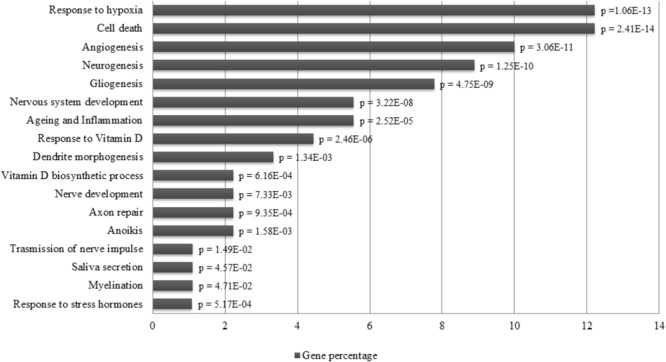
Pathway enrichment analysis of miRNAs. Over-represented biological functions of let-7i-5p, miR-27b-3p, miR-142-3p, miR-107, miR-135b-5p computed by analyzing their validated molecular targets through FatiGo and David tools. On the left of the histogram are reported the over represented pathways, while, on the right the corresponding *p*-values of Fisher’s Exact Test. On the *x*-axis are reported the percentages of gene targets associated to biological functions.

The different distribution of these miRNAs in saliva of concussed athletes is most likely a systemic consequence of the different physiopathology of this typology of trauma. In order to evaluate the biological functions of DE-miRNAs, we computationally searched their validated or predicted targets. Gene ontologies and pathway associations of miRNA targets were analyzed by FatiGo and DAVID. This analysis showed that they could be involved in important biological processes related to trauma (i.e., response to hypoxia, cell death, neurogenesis, axon repair, myelination) (**Figure 6**). By screening the Human miRNA tissue atlas, we found that all validated DE-miRNAs, with exclusion of miR-142-3p, were expressed in almost all body tissues, but more abundant in brain (**Supplementary Figure S1**).

### Sperman Correlation Analysis and Paired Comparisons With Neuropsychometric Tests

**FIGURE 7:**
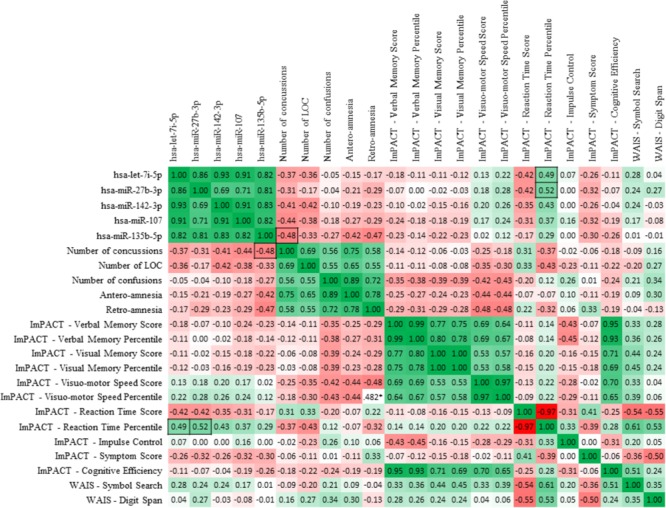
Sperman correlation of the 5 candidate miRNA biomarkers with neurocognitive assessment tools. Spearman *R*-values depict correlations between salivary concentrations of the 5 miRNAs of interest and the indexes of ImPACT test and WAIS symbol search and digit span results. Boxes highlight correlations between miRNA levels and clinical indexes which are statistically significant (*p* < 0.05). Values highlighted with black borders show the statistically significant (*p* < 0.05) correlations between miRNA levels and clinical indexes.

A summary of the ImPACT and WAIS data obtained from the concussed and non-concussed groups is illustrated in **Table 3**. Sperman correlation analysis showed a positive relationship between the ImPACT reaction time percentile and the level of microRNA let-7i-5p (*R*, 0.49; FRD 0.02) and miR-27b-3p (*R*, 0.52; FDR 0.02). In addition an inverse correlation was detected between the level of miR-135b-5p and the number of concussions (*R*, -0.48; FDR 0.05) (**Figure 7**).

**Table 3:** A summary of ImPACT (percentile score with regard to all ImPACT test takers) and WAIS data.

ImPACT domain percentile	Verbal memory	Visual memory	Motor speed	Reaction time	Symptoms score	Cognitive efficiency index	WAIS symbol search score
**Mean/Median**							
Concussed	59.8%/67%	48%/47%	54.6%/55.5%	55.6%/72.5%	18.8%/4%	0.41%/0.4%	37.1/36.5
Non-concussed	11%/72.5%	69.4%/66.5%	50.9%/57%	41.1%/38%	2.63%/1%	0.26%/0.3%	39.1/40

No statistically significant difference was seen in the ImPACT cognitive efficiency index (summary composite score of all sub domains), or WAIS symbol search score (Mann–Whitney *U, p* = 0.54 and 0.204, respectively) between the concussed and non-concussed group.

### Single TaqMan Assay at Later Time Points

In order to assess the progress or decline of the selected miRNAs at later time points from concussion, we subsequently tested the expression of the 5 selected miRNAs in a separate and independent group, comprising 12 concussed athletes. Expression changes were compared with early concussed athletes (48–72 h) and non-concussed athletes. Results showed in **Supplementary Figure S2** confirmed a *p*-value < 0.05 between the non-concussed athletes and concussed athletes at early time points. Specifically, miR-27b-3p (*p* = 0.053), let-7i-5p (*p* = 0.014), miR-142-3p (*p* = 0.028), miR-107 (*p* = 0.015), miR-135b-5p (*p* = 0.042).

An additional significant result was found in the fold change of let-7i-5p (*p* = 0.036) between concussed athletes early time point (48–72 h) and concussed athletes later time point (>120 h). No others significant results were found across the groups.

## Discussion

Despite the millions of sports-related concussions that occur annually, currently there are no available and sufficiently sensitive molecular-biomarkers to make a clear diagnosis of concussion, to predict recovery and an athlete’s readiness to return to play.

Among the most studied T-tau, NFL in CSF (Hesse et al., 2000; Nylen et al., 2006; Zetterberg et al., 2006; Neselius et al., 2012), S-100β, GFAP and its breakdown products, and UCHL-L1 in plasma/serum, but their utility in the diagnosis of mild to moderate TBI is still unknown (Vos et al., 2010; Brophy et al., 2011; Papa et al., 2012a,b; Thelin et al., 2013; Di Pietro et al., 2015) and in the majority of the cases their use is limited to alteration of BBB (Zetterberg et al., 2013).

Areas under curve of the most studied protein biomarkers in TBI are presented in **Supplementary Table S3** as previously reported by Di Pietro et al. (2018).

In this study, we examined saliva, a novel fluid for discovering new biomarkers within this context. The potential of saliva as a biomarker of mTBI has been increasingly recognized in recent years (Hicks et al., 2018; Johnson et al., 2018). A variety of molecular and microbial analytes (Aas et al., 2005; Park et al., 2006; Bonne and Wong, 2012) have been identified in saliva and considering that salivary glands are highly permeable and enveloped by capillaries, these molecules have the potential to mirror the blood content and diagnose systemic disorders (Burbelo et al., 2012).

Our results showed for the majority of the detected salivary proteins, a moderate but not statistically significant downregulation in concussed athletes. We would interpret this as a programmed shut-down of the synthesis of proteins that are not required following injury but the interplay between different cellular pathways makes the interpretation somewhat speculative. However, this downregulation is in agreement with our previous findings (Di Pietro et al., 2013). In this previous study, we were able to demonstrate, using an *in vitro* stretch model of mild TBI, that mTBI triggers a controlled gene program and a hypometabolic state, as an adaptive response finalized to neuroprotection, similar to that found in hibernators and in ischemic preconditioning.

On the contrary, our results show a different and significant expression of microRNAs in the two groups. MiRNAs are a quite recently discovered class of non-coding RNAs, which plays key roles in the regulation of gene expression. They are found in every human tissue and biofluid, are resistant to RNAse degradation and have the ability to cross the BBB (Fire et al., 1998; Chan et al., 2005; Lim et al., 2005; Gauthier and Wollheim, 2006; Bi et al., 2009; Friedman et al., 2009; Li and He, 2012; Szafranski et al., 2015).

MiRNAs are attracting increasing interest in clinical research as potential biomarkers for the detection, identification and classification of cancers and other disease states including neurodegenerative diseases and most recently, brain trauma (Redell et al., 2010; Vallelunga et al., 2014; Ragusa et al., 2016, 2017; Di Pietro et al., 2017).

The 5 upregulated microRNAs found in this study, are not brain-specific but they are also expressed in other tissues such as heart, kidney, or testis, as described in two studies aimed to build comprehensive human miRNA tissue atlas catalog and annotating accurate sequence, expression and conservation information for the large number of recently proposed miRNAs (Landgraf et al., 2007; Ludwig et al., 2016). However, 2 of them were described in the context of TBI as potential biomarkers in other biofluids. In particular, miR-27b was described in serum (Bhomia et al., 2016) and CSF (Yang et al., 2016) and miR-142-3p was identified in plasma of patients mildly injured and at greater risk of developing amnesia and therefore, post-concussive syndromes (Mitra et al., 2017).

In addition, other animal studies have described the involvement of these miRs in TBI research.

MiR-27b was found overexpressed in mouse neurons, inhibiting neuronal apoptosis induced by intrauterine hypoxia (Chen et al., 2014). MiR-27a was also found rapidly downregulated in injured cortex and this change coincided with increased expression of the proapoptotic Bcl-2 family members such as Noxa, Puma, and Bax (Sabirzhanov et al., 2014).

MicroRNA let-7i was found in both serum and cerebrospinal fluid immediately after blast wave exposure. In addition, this miRNA plays a role in the regulatory pathways of several inflammatory cytokines and therefore and ideal candidate biomarkers in TBI (Balakathiresan et al., 2012).

Finally, miR-107 regulates granulin/progranulin with implications for traumatic brain injury and neurodegenerative disease (Wang et al., 2010).

According to our results two miRNAs, let-7i-5p and miR-27b-3p, positively correlate with the ImPACT reaction time percentile. This finding suggests that these miRNAs contribute to the process of recovery, enabling natural mechanisms of neuroprotection (Balakathiresan et al., 2012; Chen et al., 2014; Sabirzhanov et al., 2014) rather than providing a biomarker of biological damage. Interestingly, no information is available about the function of miR-135b-5p, which is negatively correlated with the number of concussions. Further studies will be required to further elucidate the nature and mechanisms of this miRNA in mTBI. Notably, we found a general upregulation of miRNAs in mTBI patients: we could speculate that the increased release of glutamate observed after concussion can induce an excitatory stimulus on parasympathetic neurons that innervate the submandibular and sublingual salivary glands (Mitoh et al., 2004; Guerriero et al., 2015). This should lead to an increased saliva secretion and, theoretically, to a similar effect on miRNA release.

The fact that neither our concussed or non-concussed cohort had statistically different scores in the ImPACT of WAIS assessment may potentially indicate that levels of expressed miRNAs may serve as a more sensitive test to resolve a concussive event. Although possibly useful within the clinical management of concussion, multiple publications have demonstrated that these assessments have significant limitations when used in isolation to diagnose/resolve a concussive event (Schatz et al., 2006; Broglio et al., 2007).

Finally, the computational analysis of the present study identified that miRNAs contribute to multiple CNS processes, such as neurogenesis, and axon repair and myelination, providing evidence for further link miRNAs to mechanisms that are widely associated with concussion symptoms and recovery. Incidentally, the comparison among the whole set of inflammatory proteins studied and targets of five DE-miRNAs, showed only about 5% overlap. This may suggest that molecular circuits regulated by DE-miRNAs are quite unrelated to classical inflammation pathways.

Univariate ROC curves showed a good diagnostic accuracy for all the five miRNAs identified in this study, supporting the potential use of these biomarkers in clinical decision-making in sport and military medicine. Specifically, the goal of using miRNA biomarkers would be to detect mTBI with the highest possible accuracy, and a single biomarker often could be not sufficient. For this reason, the use of a combination of biomarkers is preferable, as it should increase diagnostic accuracy. However, by using a multivariate ROC curve model considering all the 5 miRNAs, we did not observe a significant improvement of diagnostic performances with respect to the univariate ROC curves. The comparison of performances of all classification models showed that let-7i-5p is the best classifier.

In conclusion, this study explored a suite of salivary miRNA based biomarkers for diagnosis of concussion. Five biomarkers were identified with potential utility to distinguish concussed athletes from non-concussed athletes after 48–72 h from injury. In addition, preliminary results of later time points (>120 h), showed that these miRs are not able to discriminate concussed from non-concussed athletes.

## Study Limitations

Several limitations of this study must be considered.

In the first instance, the lack of miRNA validation. Only 5 of the 21 miRs selected in the discovery study, which means <25% of candidates, were confirmed in the validation cohort of the 22 athletes analyzed. This can be due to several factors; the sample size for example, although partially justified with the power analysis, is smaller than existing microRNA studies (Di Pietro et al., 2017; Johnson et al., 2018). Secondly, cohort of patients was enrolled within a broad sampling time window (48–72 h), which represents a long interval considering the biological mechanism of microRNAs and their role as molecular regulators. In addition, we are not aware if miRNA expression varies at specific conditions such as: fasting or circadian rhythm.

Currently, the majority of the studies on miRNA biomarkers, reports the comparison between miRNAs identified in patients and in healthy controls without testing other factors that impact the miRNA abundance. For this reason, the evaluation of miRNA biomarkers in terms of stability and variability in a population of individuals without known diseases, remains one of the main challenges. Thus, variable markers can only be used if there are substantial differences in the signal between affected and unaffected individuals.

Another limitation of the study is that long-term outcome including return to play data and chronic symptomatology in these patients was not collected. Finally, we do not know how soon these biomarkers became upregulated in saliva after concussion.

Further research, including an earlier and detailed time of collection together with an enlarged group of study and a comparison with internal controls such as within the same subjects before and after concussion, or with orthopedic injury population and an additional study showing microRNA variation after exercise, is needed in order to aid the diagnosis of concussion in the treatment room, clinic and possibly pitch-side.

However, the most important finding showed in this paper, is that saliva miRNA outperforms saliva protein for identifying mTBI and provides support for the idea that peripheral microRNA patterns hold promise where years of protein biomarker work have fallen.

## Author Contributions

VD conception and design of the study, acquisition and analysis of data, and drafting the manuscript or figures. EP, MR, CB, DD, MF, and CD contributed to the data acquisition and analysis. AL, MP, MG, DH, VS, AKB, and AB conception and design of the study and editing the manuscript or figures. All authors approved the final version.

## Conflict of Interest Statement

The University of Birmingham has intellectual property associated with miRNA listed in this manuscript. The authors declare that the research was conducted in the absence of any commercial or financial relationships that could be construed as a potential conflict of interest.

## Footnotes

1 https://www.olink.com/

2 http://www.tm4.org

3 http://carolina.imis.athena-innovation.gr/diana_tools/web/index.php

4 http://mirtarbase.mbc.nctu.edu.tw

5 babelomics.bioinfo.cipf.es

6 https://david.ncifcrf.gov

7 https://ccb-web.cs.uni-saarland.de/tissueatlas/

8 http://www.metaboanalyst.ca/

## Supplementary Material

FIGURE S1 Human miRNA tissue atlas. Expression of the 5 miRNAs in different human tissue.

FIGURE S2 Boxplot of the 5 candidate miRNA biomarkers at early and late time points. Boxplot comparing the relative expression of the 5 microRNAs at different time points, 48–72 h and >120 h of concussed athletes to non-concussed athletes. ANOVA test was used to show significant results (*p* < 0.05) across groups.

TABLE S1 List of biomarkers analyzed with the Proseek proximity extension assay.

TABLE S2 *t*-Test for the 65 proteins detected in saliva of non-concussed and concussed athletes.

TABLE S3 Area under the curve (AUC) of representative TBI biomarkers.

## Abbreviations

AUC: area under the curve
BBB: brain–blood barrier
CNS: central nervous system
CSF: cerebral spinal fluid
CTE: chronic traumatic encephalopathy
DE-miRNAs: differentially expressed microRNAs
FDA: food and drug administration
FDR: false discovery rate
GFAP: glial fibrillary acid protein
GMN: global median normalization
GO: gene ontology
ImPACT: Immediate Post-Concussion Assessment and Cognitive Testing
KEGG: Kyoto encyclopedia of genes and genomes
miRs or miRNAs: microRNAs
mTBI: mild traumatic brain injuries
NAB: neuropsychological assessment battery
NFL: neurofilament light
NPX: normalized protein expression units
POC: point-of-care
ROC: operating characteristic
SAM: significance of microarrays analysis
SCAT3: sport concussion assessment tool
TBI: traumatic brain injuries
T-tau: Total tau
UCHL-L1: ubiquitin carboxyl-terminal esterase L1
WAIS-IV: Wechsler Adult Intelligence Scale-Fourth Edition
