# Supplementary figures and images for: Salivary MicroRNAs: Diagnostic Markers of Mild Traumatic Brain Injury in Contact-Sport
Source: Front Mol Neurosci. 2018 Aug 20;11:290. doi: 10.3389/fnmol.2018.00290 (PMC6109773; doi:10.3389/fnmol.2018.00290)

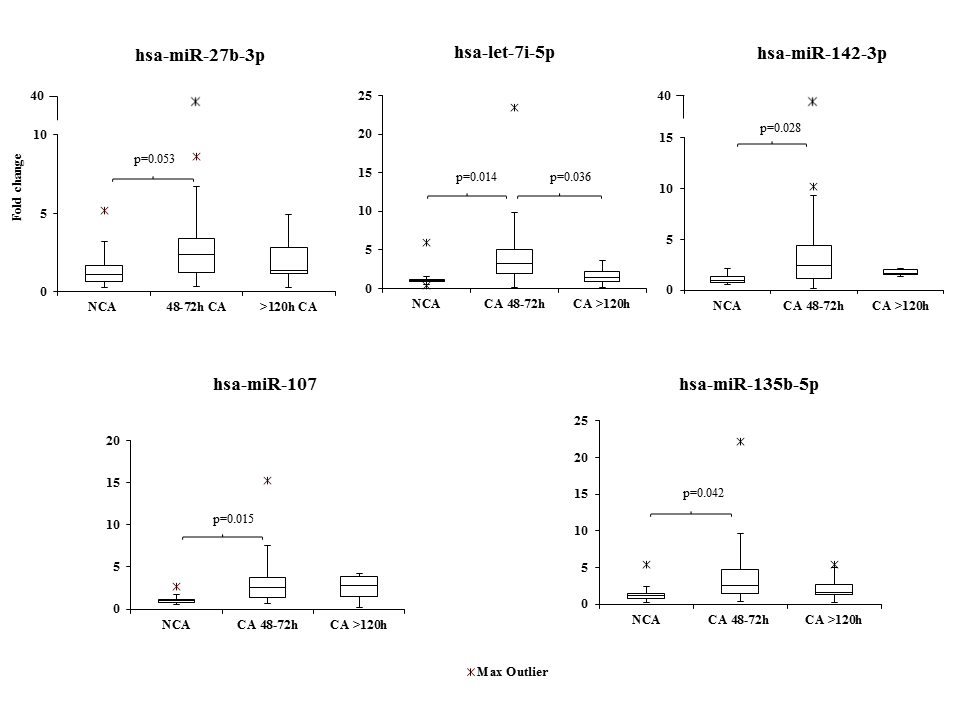

Supplement: FIGURE S2 — Boxplot of the 5 candidate miRNA biomarkers at early and late time points. Boxplot comparing the relative expression of the 5 microRNAs at different time points, 48–72 h and >120 h of concussed athletes to non-concussed athletes. ANOVA test was used to show significant results (p < 0.05) across groups. [file Image_2.TIF]
