# Supplementary material for: Salivary MicroRNAs: Diagnostic Markers of Mild Traumatic Brain Injury in Contact-Sport
Source: Front Mol Neurosci. 2018 Aug 20;11:290. doi: 10.3389/fnmol.2018.00290 (PMC6109773; doi:10.3389/fnmol.2018.00290)
Supplement: TABLE S1 — List of biomarkers analyzed with the Proseek proximity extension assay. [file Data_Sheet_1.docx]

**Supplentary Table 1:** List of biomarkers analysed with the Proseek proximity extension assay

| **Biomarker** | | |
| --- | --- | --- |
| Adenosine Deaminase (ADA) | Interleukin-18 (IL-18) | Signaling lymphocytic activation molecule (SLAMF1) |
| Artemin (ARTN) | Interleukin-18 receptor 1 (IL-18R1) | SIR2-like protein 2 (SIRT2) |
| Axin-1 (AXIN1) | Interleukin-2 (IL-2) | STAM-binding protein (STAMPB) |
| Beta-nerve growth factor (Beta-NGF) | Interleukin-2 receptor subunit beta (IL-2RB) | Stem cell factor (SCF) |
| Brain-derived neurotrophic factor (BDNF) | Interleukin-20 (IL-20) | Sulfotransferase 1A1 (ST1A1) |
| C-C motif chemokine 19 (CCL19) | Interleukin-20 receptor subunit alpha (IL-20RA) | T cell surface glycoprotein CD6 isoform (CD6) |
| C-C motif chemokine 20 (CCL20) | Interleukin-22 receptor subunit alpha-1 (IL-22 RA1) | T-cell surface glycoprotein CD5 (CD5) |
| C-C motif chemokine 23 (CCL23) | Interleukin-24 (IL-24) | Thymic stromal lymphopoietin (TSLP) |
| C-C motif chemokine 28 (CCL28) | Interleukin-33 (IL-33) | Thymic stromal lymphopoietin (TSLP) |
| CD40L receptor (CD40) | Interleukin-4 (IL-4) | TNF-beta (TNFB) |
| CUB domain-containing protein 1 (CDCP1) | Interleukin-5 (IL-5) | TNF-related activation-induced cytokine (TRANCE) |
| Cystatin D (CST5) | Interleukin-6 (IL-6) | TNF-related activation-induced cytokine (TRANCE) |
| Delta and Notch-like epidermal growth factor-related receptor (DNER) | Interleukin-7 (IL-7) | TNF-related apoptosis-inducing ligand (TRAIL) |
| Eotaxin-1 (CCL11) | Interleukin-8 (IL-8) | Transforming growth factor alpha (TGF-alpha) |
| Eukaryotic translation initiation factor 4E-binding protein 1 (4E-BP1) | Latency-associated peptide transforming growth factor beta 1 (LAP TGF-beta-1) | Tumor necrosis factor (Ligand) superfamily, member 12 (TWEAK) |
| Fibroblast growth factor 19 (FGF-19) | Leukemia inhibitory factor (LIF) | Tumor necrosis factor (TNF) |
| Fibroblast growth factor 21 (FGF-21) | Leukemia inhibitory factor receptor (LIF-R) | Tumor necrosis factor ligand superfamily member 14 (TNFSF14) |
| Fibroblast growth factor 5 (FGF-5) | Macrophage colony-stimulating factor 1 (CSF-1) | Tumor necrosis factor receptor superfamily member 9 (TNFRSF9) |
| Fms-related tyrosine kinase 3 ligand (Flt3L) | Macrophage inflammatory protein 1-alpha (MIP-1 alpha ) | Urokinase-type plasminogen activator (uPA) |
| Fractalkine (CX3CL1 ) | Matrix metalloproteinase-1 (MMP-1) | Vascular endothelial growth factor A (VEGF-A) |
| Glial cell line-derived neurotrophic factor (hGDNF) | Matrix metalloproteinase-10 (MMP-10) |  |
| Hepatocyte growth factor (HGF) | Monocyte chemotactic protein 1 (MCP-1) |  |
| Interferon gamma (IFN-gamma) | Monocyte chemotactic protein 2 (MCP-2) |  |
| Interleukin-1 alpha (IL-1 alpha) | Monocyte chemotactic protein 3 (MCP-3) |  |
| Interleukin-10 (IL-10) | Monocyte chemotactic protein 4 (MCP-4) |  |
| Interleukin-10 receptor subunit alpha (IL-10RA) | Natural killer cell receptor 2B4 (CD244) |  |
| Interleukin-10 receptor subunit beta (IL-10RB) | Neurotrophin-3 (NT-3) |  |
| Interleukin-12 subunit beta (IL-12B) | Neurturin (NRTN) |  |
| Interleukin-13 (IL-13) | Oncostatin-M (OSM) |  |
| Interleukin-15 receptor subunit alpha (IL-15RA) | Osteoprotegerin (OPG) |  |
| Interleukin-17A (IL-17A) | Programmed cell death 1 ligand 1 (PD-L1) |  |
| Interleukin-17C (IL-17C) | Protein S100-A12 (EN-RAGE ) |  |

**Supplementary Table 2**: T-test for the 65 proteins detected in saliva of non-concussed and concussed athletes.

Test was applied for proteins detected in more than 75% of the samples (65 proteins in total and listed below). P-values were adjusted for multiple testing using the False Discovery Rate method of correlation (FDR). None of the proteins listed below was found significantly different in the two groups. P-values, adjusted p-values, differences and fold changes, average and standard deviation for the 2 groups are listed below.

| **Protein** | **Adjusted p-value** | **P-value** | **Difference**  **between the 2 groups** | **Fold change** | **Non concussed athletes average** | **Standard deviation** | **Concussed athletes average** | **Standard deviation** |
| --- | --- | --- | --- | --- | --- | --- | --- | --- |
| NT.3 | 2.32E-01 | 3.57E-03 | -1.06 | -2.08 | 2.23 | 0.76 | 1.18 | 0.53 |
| ARTN | 9.38E-01 | 3.84E-02 | -0.73 | -1.66 | 2.33 | 0.65 | 1.61 | 0.73 |
| IL.20RA | 9.38E-01 | 8.53E-02 | 0.25 | 1.19 | 0.30 | 0.21 | 0.60 | 0.41 |
| IL.12B | 9.38E-01 | 9.92E-02 | -0.92 | -1.89 | 2.72 | 1.32 | 1.80 | 0.84 |
| EN.RAGE | 9.38E-01 | 2.46E-01 | -0.24 | -1.18 | 7.79 | 0.39 | 7.55 | 0.45 |
| 4E.BP1 | 9.38E-01 | 2.59E-01 | 0.70 | 1.63 | 7.01 | 1.38 | 7.71 | 1.17 |
| uPA | 9.38E-01 | 3.03E-01 | -0.50 | -1.41 | 10.44 | 0.96 | 9.94 | 1.04 |
| IL.8 | 9.38E-01 | 3.28E-01 | -0.40 | -1.32 | 13.17 | 0.70 | 12.77 | 0.91 |
| LAP.TGF.beta.1 | 9.38E-01 | 3.51E-01 | -0.48 | -1.39 | 5.50 | 1.24 | 5.02 | 0.85 |
| TNFSF14 | 9.38E-01 | 3.58E-01 | -0.58 | -1.49 | 7.95 | 1.39 | 7.37 | 1.20 |
| CXCL1 | 9.38E-01 | 3.69E-01 | -0.48 | -1.40 | 12.03 | 0.64 | 11.54 | 1.42 |
| TRAIL | 9.38E-01 | 3.82E-01 | 0.27 | 1.21 | 10.17 | 0.38 | 10.44 | 0.81 |
| Flt3L | 9.38E-01 | 3.84E-01 | 0.30 | 1.23 | 2.87 | 0.66 | 3.17 | 0.74 |
| CCL20 | 9.38E-01 | 4.17E-01 | -0.77 | -1.70 | 6.77 | 0.78 | 6.00 | 2.62 |
| HGF | 9.38E-01 | 4.46E-01 | -0.40 | -1.32 | 8.62 | 1.26 | 8.22 | 0.91 |
| OPG | 9.38E-01 | 4.49E-01 | -0.30 | -1.23 | 9.69 | 0.45 | 9.39 | 1.05 |
| LIF.R | 9.38E-01 | 4.56E-01 | -0.17 | -1.13 | 5.21 | 0.43 | 5.04 | 0.49 |
| VEGF.A | 9.38E-01 | 4.73E-01 | -0.12 | -1.08 | 13.34 | 0.21 | 13.22 | 0.40 |
| TNFRSF9 | 9.38E-01 | 4.82E-01 | -0.35 | -1.28 | 4.25 | 0.96 | 3.90 | 1.12 |
| AXIN1 | 9.38E-01 | 4.93E-01 | -0.22 | -1.16 | 1.65 | 0.77 | 1.43 | 0.81 |
| CST5 | 9.38E-01 | 5.01E-01 | 0.23 | 1.17 | 7.71 | 0.63 | 7.94 | 0.78 |
| CSF.1 | 9.38E-01 | 5.11E-01 | -0.28 | -1.22 | 7.43 | 0.99 | 7.15 | 0.79 |
| CXCL10 | 9.38E-01 | 5.16E-01 | 0.79 | 1.72 | 9.33 | 2.95 | 10.12 | 1.98 |
| MMP.1 | 9.38E-01 | 5.19E-01 | -0.40 | -1.32 | 10.36 | 0.97 | 9.96 | 1.53 |
| MCP.1 | 9.38E-01 | 5.20E-01 | -0.38 | -1.30 | 11.20 | 1.04 | 10.82 | 1.39 |
| TGF.alpha | 9.38E-01 | 5.34E-01 | 0.24 | 1.18 | 5.48 | 0.54 | 5.72 | 0.97 |
| CXCL11 | 9.38E-01 | 5.34E-01 | 0.52 | 1.43 | 5.10 | 1.75 | 5.62 | 1.73 |
| SIRT2 | 9.38E-01 | 5.40E-01 | -0.43 | -1.34 | 4.43 | 1.54 | 4.00 | 1.35 |
| ST1A1 | 9.38E-01 | 5.48E-01 | -0.43 | -1.34 | 4.54 | 1.52 | 4.11 | 1.44 |
| CCL28 | 9.38E-01 | 5.51E-01 | -0.27 | -1.21 | 7.75 | 0.57 | 7.48 | 1.21 |
| OSM | 9.38E-01 | 5.51E-01 | -0.36 | -1.28 | 8.18 | 1.17 | 7.82 | 1.34 |
| SCF | 9.38E-01 | 5.68E-01 | 0.19 | 1.14 | 3.56 | 0.56 | 3.75 | 0.79 |
| DNER | 9.38E-01 | 5.68E-01 | 0.03 | 1.02 | 10.30 | 0.08 | 10.33 | 0.12 |
| CD6 | 9.38E-01 | 5.76E-01 | -0.25 | -1.19 | 1.65 | 1.36 | 1.40 | 0.88 |
| IL.10 | 9.38E-01 | 5.78E-01 | -0.18 | -1.13 | 1.73 | 0.59 | 1.28 | 1.03 |
| TRANCE | 9.38E-01 | 5.86E-01 | -0.29 | -1.22 | 2.45 | 1.29 | 2.16 | 1.23 |
| TWEAK | 9.38E-01 | 6.24E-01 | -0.29 | -1.23 | 7.55 | 1.15 | 7.26 | 1.35 |
| LIF | 9.38E-01 | 6.27E-01 | -0.19 | -1.14 | 2.72 | 0.69 | 2.53 | 0.92 |
| IL.7 | 9.38E-01 | 6.44E-01 | 0.22 | 1.17 | 4.06 | 0.38 | 4.28 | 1.34 |
| IL.18R1 | 9.38E-01 | 6.49E-01 | -0.15 | -1.11 | 8.30 | 0.64 | 8.15 | 0.75 |
| IL.17A | 9.38E-01 | 6.51E-01 | -0.21 | -1.16 | 1.43 | 1.15 | 1.22 | 1.03 |
| IL.1.alpha | 9.38E-01 | 6.53E-01 | -0.20 | -1.15 | 10.52 | 0.90 | 10.32 | 0.93 |
| CCL4 | 9.38E-01 | 6.73E-01 | -0.33 | -1.26 | 4.88 | 1.15 | 4.55 | 1.98 |
| CCL23 | 9.38E-01 | 6.86E-01 | -0.15 | -1.11 | 0.31 | 0.36 | 1.95 | 0.98 |
| CXCL6 | 9.38E-01 | 7.12E-01 | -0.28 | -1.21 | 8.19 | 0.74 | 7.91 | 2.08 |
| CDCP1 | 9.38E-01 | 7.13E-01 | 0.17 | 1.13 | 6.80 | 1.02 | 6.98 | 0.92 |
| CXCL9 | 9.38E-01 | 7.39E-01 | -0.18 | -1.13 | 8.51 | 0.96 | 8.33 | 1.24 |
| MIP.1.alpha | 9.38E-01 | 7.39E-01 | -0.21 | -1.16 | 3.96 | 0.70 | 3.65 | 1.91 |
| CXCL5 | 9.38E-01 | 7.58E-01 | -0.30 | -1.23 | 11.70 | 1.59 | 11.40 | 2.43 |
| IL.18 | 9.38E-01 | 7.61E-01 | 0.16 | 1.12 | 9.79 | 1.07 | 9.95 | 1.10 |
| IL.6 | 9.38E-01 | 7.70E-01 | 0.15 | 1.11 | 4.34 | 1.06 | 4.49 | 1.11 |
| PD.L1 | 9.38E-01 | 7.71E-01 | 0.08 | 1.06 | 1.95 | 0.77 | 2.18 | 0.72 |
| CD40 | 9.38E-01 | 7.88E-01 | 0.10 | 1.07 | 9.97 | 0.65 | 10.07 | 0.85 |
| IL.10RB | 9.38E-01 | 8.01E-01 | 0.06 | 1.05 | 4.65 | 0.43 | 4.71 | 0.62 |
| CD5 | 9.38E-01 | 8.13E-01 | -0.09 | -1.06 | 5.06 | 0.72 | 4.97 | 0.86 |
| MCP.2 | 9.38E-01 | 8.17E-01 | -0.15 | -1.11 | 3.98 | 1.12 | 3.83 | 1.49 |
| CCL11 | 9.38E-01 | 8.23E-01 | 0.10 | 1.07 | 2.25 | 0.40 | 2.23 | 1.44 |
| TNFB | 9.78E-01 | 8.73E-01 | -0.04 | -1.03 | 0.81 | 0.47 | 0.69 | 0.68 |
| FGF.19 | 9.82E-01 | 9.15E-01 | -0.04 | -1.03 | 1.48 | 0.71 | 1.45 | 0.72 |
| ADA | 9.82E-01 | 9.31E-01 | -0.04 | -1.03 | 5.77 | 1.07 | 5.73 | 1.06 |
| MMP.10 | 9.82E-01 | 9.35E-01 | 0.07 | 1.05 | 5.52 | 1.68 | 5.59 | 1.82 |
| CASP.8 | 9.82E-01 | 9.40E-01 | -0.05 | -1.03 | 7.49 | 1.42 | 7.44 | 1.34 |
| STAMPB | 9.82E-01 | 9.54E-01 | -0.03 | -1.02 | 4.87 | 1.32 | 4.84 | 1.07 |
| CX3CL1 | 9.82E-01 | 9.77E-01 | -0.01 | -1.01 | 7.59 | 0.33 | 7.58 | 1.10 |
| CCL19 | 9.82E-01 | 9.82E-01 | -0.02 | -1.02 | 4.49 | 1.64 | 4.47 | 2.53 |
|  |  |  |  |  |  |  |  |  |

**Supplementary Table 3:** Area under the curve (AUC) of representative TBI biomarkers

| **Biomarkers** | **AUC** | **Cohort** | **Condition** | **N** | **Controls** | **Reference** | **Timing** | **Comment** |
| --- | --- | --- | --- | --- | --- | --- | --- | --- |
| S100B | 0.87 | TBI  all severity | TBI  vs  non-TBI | 50 | 50 | Borg K et al., 2012 | within 6h | non-specific |
| S100B | 0.68 | mTBI | ice hockey vs  pre-season | 28 | 28 | Shahim P,  2014 | within 1h | poor performance |
| NSE | 0.82 | TBI  all severity | TBI  vs  non-TBI | 50 | 50 | Borg K et al., 2012 | within 6h | non-specific |
| NSE | 0.54 | mTBI |  | 28 | 28 | Shahim P,  2014 | within 1h | poor performance |
| NSE | 0.64 | mTBI | clinically important injury | 25 | 82 | Wolf H, 2013 | day 1 | non-specific |
| Myelin-basic protein | 0.66 | TBI  all severity | TBI  vs  non-TBI | 50 | 50 | Borg K et al., 2012 | within 6h | poor performance |
| Cleaved Tau | 0.74 | mTBI | injury  vs  pre-season | 28 | 28 | Siman R, 2015 | At 36h | late |
| Total Tau | 0.8 | mTBI | Ice hockey vs  pre-season | 28 | 28 | Shahim P, 2014 | within 1h | promising |
| GFAP | 0.84 | mild-moderate TBI | positive CT | 209 | 188 | Papa L et al, 2014 | at 4h | limited sensitivity |
| UCH-L1 | 0.87 | mTBI | GCS 15  Vs  controls | 86 | 199 | Papa L,  2012 | within 1h | promising |
| UCH-L1 | 0.73 | TBI | positive CT | N/A | 199 | Papa L,  2012 | within 1h | promising |
| Amyloid-β | N/A | sTBI | TBI  vs  controls | 12 | 20 | Mondello S,  2014 | day 1 | poor sensitivity |
| All-Spectrin break-down | 0.76 | mTBI | injury  vs  pre-season | 25 | N/A | Siman R, 2015 | At 36h | late |
| CTS5 | N/A | TBI  all severity | sTBI  vs  orthopedic injury | 30 | 30 | Hill JL, 2017 | within 1h | promising |
